# Well-Being as Human Development, Equality, Happiness and the Role of Freedom, Activism, Decentralization, Volunteerism and Voter Participation: A Global Country-Level Study

**DOI:** 10.3389/fpsyg.2021.745818

**Published:** 2021-09-17

**Authors:** Douglas D. Perkins, Mehmet Reha Ozgurer, Andrew Lupton, Shadi Omidvar-Tehrani

**Affiliations:** Department of Human and Organizational Development, Peabody College, Vanderbilt University, Nashville, TN, United States

**Keywords:** well-being, human development, inequality, happiness, freedom, fiscal and political decentralization, voluntarism, voter participation

## Abstract

We propose and test a new model for predicting multiple quantitative measures of well-being globally at the country level based on the United Nations Human Development Index (HDI), income inequality (Net Gini), and National Happiness Index (NHI; U.N. Sustainable Development Solutions Network world survey of life satisfaction). HDI consists of per-capita Gross National Income (economic well-being), average life expectancy (proxy for health well-being), and educational attainment (capabilities well-being). Using data on 105 countries representing 95% of the world’s population, a history of grassroots activism (Global Non-violent Action Database), civil liberties and political rights (Freedom Score), political and fiscal decentralization, and voter participation (Institute for Democracy and Electoral Assistance) correlate with HDI and NHI. Citizen volunteering (Gallup Civic Engagement Index) predicts only NHI. In multivariate analyses, Freedom Score is the most robust predictor of all well-being measures, including income equality. Fiscal decentralization and voter turnout also predict HDI and NHI, controlling for other influences. Based on prior analyses in the Global Development of Applied Community Studies project, implications and recommendations are discussed for developing community human research and professional resources across 12 disciplines in countries where they are needed based on social justice, citizenship, well-being, inequality, human rights, and other development challenges. We recommend individual and community-level and qualitative analyses of the above predictors’ relationships with these same conceptualizations of well-being, as well as consideration of other social, cultural and political variables and their effect on well-being.

## Indicators of National Well-Being

Famously, what counts for economists is whatever one can measure quantitatively, and especially monetarily. Historically, per-capita Gross Domestic Product (GDP; the market value of all goods and services produced in a country in a specified period, e.g., annually) was the dominant measure of national well-being and vitality used, not only by economists, but by international security, development, and political institutions and experts. However, critiques of the limitations of GDP as a measure even of sustainable economic development began decades ago (Giannetti et al., 2015). For example, Gross National Income (GNI), which consists of a country’s GDP plus incomes earned in that country by foreign residents minus income earned in the country by non-residents, was created to measure just the income derived from GDP for residents (removing income from direct foreign investment that leaves the country). More importantly, non-economic indicators of well-being on a national scale were needed. Various alternative concepts and measures have been proposed, many focusing on broader conceptions of human and community “social development” (Sen, 1999, 2008).

We begin by identifying three of the most widely used measures of national well-being and then explore several political, citizenship-related, and social justice predictors in order to create and test a model of well-being globally at the societal level. Well-being at the individual level is multidimensional and complicated enough; measuring it at the national level is certainly no less complex. Each measure of well-being emphasizes one or multiple different aspects of well-being, as explained below. Consideration and testing of each dimension and measure of national well-being is essential to establish both content and construct validity and to improve our understanding of societal well-being and the factors associated with it. Thus our purpose is to answer the question: How well do a history of grassroots activism, political and fiscal decentralization, political rights and civil liberties, voter participation, and citizen volunteerism predict three forms and measures of national well-being: human development, income equality, and happiness?

### Human Development Index

Human Development Index was created by the United Nations Development Program to measure human development, wellness, and quality of life in a society across multiple dimensions. It consists of the mean of three components: (1) per capita GNI (as a proxy for material or economic well-being), (2) population life expectancy (as a crude proxy for general health and bodily wellness), and (3) an education index based on averaging the mean years of schooling for adults over 24 years old and expected years of schooling for school-aged children (as a crude proxy for mental development or human capabilities). Thus, HDI measures development along two social dimensions and one economic dimension with the goal of providing a slightly broader indicator based on widely available population measures. Many studies have validated the HDI by relating it to a variety of other development criteria, including GDP per capita, health expenditures, and other social and economic well-being variables (Van, 2017). Churilova et al. (2019) compared HDI with other variables of well-being and found it to be a strong indicator of human development, especially in countries with highly developed economies. Scherbov and Gietel-Basten (2020) argue that their Human Life Indicator, focusing more on just life expectancy, correlates highly with HDI, but is simpler to calculate and interpret, has fewer data requirements and less measurement error, and is more consistent over time. HDI has also been critiqued for its limitations as a comprehensive measure of population well-being and the many other dimensions it ignores (Ranis et al., 2006). However, HDI has become the most widely used and accepted international measure of development, and due to the alternatives lacking complete data, we use the HDI.

### Inequality (Gini)

The per capita GNI component of HDI assumes that what matters at the national level is simply higher income. But income *inequality* has been found to be a significant negative societal-level factor in development at the individual, community and societal levels. Wilkinson and Pickett (2009) and others have marshaled voluminous evidence that virtually every modern problem of individual well-being, family and community life—violence, drugs, crime, and mass incarceration; mental illness; poor educational outcomes, teenage births, and long working hours; obesity, other physical health problems and premature death—are all more likely to occur in a less equal society. Critically, they find that inequality effects are even greater among developed, industrialized countries, demonstrating that, while poverty clearly has negative effects on social outcomes, wealth and strong economies are not enough to ensure well-being. The most widely used indicator of income inequality is the Gini coefficient, named for the Italian statistician and sociologist who proposed it, Corrado Gini (see Measures, below). Gini has been found to predict HDI (Van, 2017).

### National Happiness Index

The final indicator explored in this study is National Happiness Index (NHI) as provided by the annual World Happiness Report (Helliwell et al., 2020). This indicator measures well-being *via* survey responses to the Gallup World Poll. This provides a more subjective, psychological perspective on national well-being and happiness, and perhaps a more accurate one as human development and wellness is self-reported. NHI has mostly been related to other factors of human development, such as the knowledge economy in Europe (Hadad, 2018). Other studies have shown the relative importance of each of the seven factors present in NHI (Carlsen, 2018). However, there has been less research into the effect of sociopolitical factors on NHI.

## Political, Social Justice and Citizenship Predictors of National Well-Being

It is essential to identify the conditions for different measures of national well-being. Prior studies have related various social and economic indicators to one of the above measures of well-being or development. For example, Van (2017) investigated the correlation of rural population, health expenditures, and other factors with HDI. But this is the first study to examine all four measures of national well-being. It is also the first to consider, not only citizenship and social justice factors, but critically also how political governance systems and structures are related to each measure of well-being.

The essential features of truly effective democratic governance systems and societies include the conduct of free and fair elections; a fair, organized and competitive party system; respecting and protecting fundamental civil liberties and human rights; and active participation of a vibrant civil society. Effective governance is linked to the well-being of nations, and these effects go beyond democracy (Helliwell, 2007). When there is less corruption and effective law, people report greater life satisfaction (Helliwell, 2003).

**FIGURE 1:**
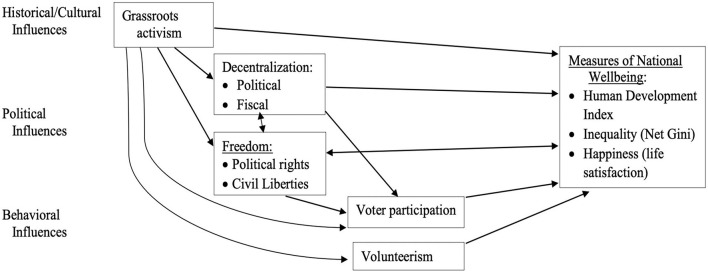
Hypothesized Political, Social Justice, and Citizenship Predictors and Measures of National Well-being.

Our country-level predictors therefore include a history of grassroots activism, the degree of government political decentralization, civil liberties and political rights, citizen volunteering, and voter participation. Those factors and outcomes constitute a new model of national well-being (see Figure 1) and next we review each of those predictor variables.

### Grassroots Activism and Well-Being

Engagement in empowering collective grassroots activism is often assumed to be related to positive mental and emotional well-being at the individual level, but the reality is less clear and more complex (Christens et al., 2013). The relationship at the national level is also still an open question. A country’s history and culture of non-violent grassroots activism is the greatest predictor of the strength of the professional and research fields of community development and community psychology (Hanitio and Perkins, 2017). Those authors argue that activism leads to youth and adult citizens who are motivated to develop knowledge and skills to study and solve community problems and activism also pressures government and higher education to support the establishment and growth of those same kinds of professional and research fields whose purpose is to improve individual and community well-being. So timing is key at both the individual and national levels: before activism helps solve major problems, or if it fails to, the relationship with well-being may be negative. Once those problems are solved, the relationship should be positive, which is what we hope to find by examining the history of activism in each country.

### Government Decentralization and Well-Being

Responsive government creates the best possible conditions for citizens’ well-being. But is centralized or decentralized government ultimately more responsive, including the supply of adequate resources to address great challenges? Voigt and Blume (2012) found a positive relationship between decentralized federalism and national happiness (or life satisfaction) across 57 countries. Fiscal, administrative, and political decentralization is the key determinant of national-local delegation and balance of power (Falleti, 2005). Government political decentralization/localization may influence development of individual and community well-being as it requires delegation of power to communities (Ahmad and Talib, 2015; Mekonnen, 2018). Frey and Stutzer (2000, 2010) argued that the relationship between democracy and happiness stems from its political process, not just the beneficial results of democracy. Their interpretation is that political decentralization leads to a closer alignment between political outcomes and voter preferences, thus improving well-being.

Supporters link the benefits of decentralization to many factors: increased political competition, reduced bureaucratic waste, improved accountability, information disclosure, strengthened democratic control, support of local minorities, policy innovation, market performance, and efficiency (Besley and Case, 1992; Kotsogiannis and Schwager, 2006).

Decentralized government is likely to be more democratic, providing more opportunities for civic space and citizen participation, resulting in the emergence of independent groups, political opposition, and for individuals to practice and experience the free choice of democratic governance (Malik and Kohli, 2013). In addition, public well-being affects governance such that a sharp decline in well-being could undermine democratic institutions (Inglehart and Klingemann, 2000).

The role of fiscal decentralization, both economically and for other dimensions of well-being may be more complicated. In a study estimating the impact of financial and political decentralization on well-being in 66 countries, local budgets and their size were critical to well-being (Bjørnskov et al., 2008). Countries with greater fiscal decentralization tend to have significantly less corruption, but that beneficial effect is dampened by regional elections and government structured by more vertical levels of administration (Altunbaş and Thornton, 2012). Somewhat paradoxically, “revenue and expenditure at the level of the subnational government are most effective in reducing corruption when those resources are nonetheless controlled by the central government” (Altunbaş and Thornton, 2012, p. 68). Another study found that decentralization increases political responsibility and political and civil liberties, but that fiscal decentralization can ultimately restrict freedom, indicating that greater accountability and political and civil rights do not necessarily lead to greater economic freedom (Bojanic, 2018a). The effect of decentralization on economic growth has been extensively studied, yet no definite conclusions have been reached, especially when developing countries are included (Bojanic, 2018b). Bardhan and Mookherjee (2000) found that decentralization can undermine governance where local authorities are “captured” by local elites.

All told, the literature suggests that political decentralization is important for national well-being, but should be accompanied by fiscal decentralization, although the evidence for the latter is mixed. Even larger than the above studies, we examine the relationship of both political and fiscal decentralization (and other predictors) with multiple dimensions of well-being in 103 countries.

### Freedom and Well-Being

According to Nobel economist Amartya Sen, freedom is “intrinsically important as the preeminent objective of development” (1999, p. 37). Implicating well-being even more directly: “Development has to be…concerned (not merely with economic growth, but) with enhancing the lives we lead and the freedoms we enjoy,” so we can become “fuller social persons, exercising our own volitions (capacities for deliberate choice) and interacting with—and influencing—the world in which we live” (Sen, 1999, p. 14–15).

Freedom—in the form of political rights and civil liberties—is fundamentally and bidirectionally related to the well-being of nations (Inglehart and Klingemann, 2000). Having more resources obviously provides individuals, families, communities, and nations with more options, hence choices, hence freedom to choose. Yet here we are interested in whether freedom, in turn, increases equality, human economic, mental and healthy development, and happiness.

Chandhoke (1995) suggests that democracy is always superior to authoritarianism for one main reason: having and exercising the fundamental rights of citizens enables them to mobilize and push the government to fulfill the promises made in the constitution and policy declarations. Of course, mobilization leads to increased participation and deepens the participation of democracy because it helps to realize the primary claim of the legitimacy of the concept—popular sovereignty. These, in turn, are factors that predict countries’ welfare, human rights, and individualism (Diener et al., 1995). Although having too many choices can undermine well-being (Schwartz, 2004), human rights and democracy appear to enhance well-being.

### Citizenship and Well-Being

#### Voter Participation

Citizenship is about citizens’ direct participation in democracy at all levels. In one study, Inglehart and Klingemann (2000) reported a robust correlation of *r* = 0.78 between the degree of democracy in countries and their level of well-being. In comparing the cantons of Switzerland, Frey and Stutzer (2000, 2010) found that those with more direct democracy (for example, more referendums and direct voting on initiatives) enjoyed higher well-being. Human rights are strongly related to voter participation in democratic societies (Bellinger, 2017). Inequality lowers voter turnout, especially among lower income quintiles (Avery, 2015). But what of voting’s effect on well-being? Voter turnout has been shown to decrease both GDP growth and income inequality (Arawatari, 2009; Mahler et al., 2014).

#### Volunteerism

Going back at least as far as Tocqueville almost 200 years ago, citizenship is also commonly, and much more frequently than voting, about volunteering in collective efforts to help one’s neighbors and improve one’s community. While most people, if they vote at all, vote just once a year or less, citizen participation in collective voluntary work in faith-based, school-based, and other community service organizations is a much more common form of citizenship. At both the individual and streetblock levels in multiple United States cities, citizen participation in community organizations is significantly related to both household economic well-being and community satisfaction and attachment (Perkins et al., 1996).

One popular formulation of volunteerism is social capital, or the resource for action within a social structure and system provided by interpersonal obligations and expectations, information channels, and social norms (Coleman, 1988). Participation in voluntary associations is often measured, and thought of, as the most common manifestation of social capital (Coleman, 1988; Helliwell, 2007). Social capital is significantly related to higher levels of subjective well-being and lower suicide rates at the national level (Helliwell, 2007). Among adolescents in a 13-country study, social capital was significantly related to well-being in the subjective forms of both place attachment/satisfaction and perceived safety (Dallago et al., 2009), which suggests the relationship with happiness or life satisfaction begins in childhood or no later than adolescence.

The direct participation of ordinary citizens in government-sanctioned policy areas is a tool to improve governance, empower citizens, promote social justice and deepen the quality of democracy. Participatory democracy is supported by the World Bank, UN-Habitat, the European Union, political parties from a wide range of ideological stripes, civil society and other non-governmental organizations, but more evidence is needed to clearly show a link with national well-being (Boulding and Wampler, 2010).

### Research Question and Hypotheses

Guided by Figure 1, our main question is: How do a history of grassroots activism, political and fiscal decentralization, political rights and civil liberties, voter participation, and citizen volunteerism predict each form and measure of national well-being in a country: (1) U.N. Human Development Index, (2) income inequality, and (3) National Happiness?

First (H1), we hypothesize that each of the above measures of well-being will be inter-correlated: HDI and Happiness positively and inequality negatively with the other two. After recoding Freedom Scores so that higher scores signify greater political rights and civil liberties, we further (H2) hypothesize that most of the independent variables in Figure 1 will be positively intercorrelated. Exceptions are that we do not have reason to expect either voter turnout or decentralization to be significantly related to volunteerism.

Focusing on our main research question above, a history and culture of grassroots activism in a country is assumed to benefit the overall well-being of its citizens because it protects against both public and private abuses of power and responds to the people’s grievances and demands. We therefore hypothesize (H3) that grassroots activism is positively related to HDI and National Happiness, and negatively related to income inequality.

The extent of decentralization, or federalism, and strength of political rights and civil liberties in a country are assumed to benefit the overall well-being of its citizens by making government more responsive to local needs, providing rights to basic government services, and protecting against physical threats and economic exploitation. We therefore hypothesize (H4) that political and fiscal decentralization and freedom scores are positively related to HDI and National Happiness Index, and negatively related to income inequality.

The level of voter turnout and civic volunteerism in a country is assumed to benefit the overall well-being of its citizens because higher voter and civic participation should both result in more of a country’s citizens’ needs being met, as voter turnout should lead to the election of politicians more responsive and aligned with voter interests and volunteerism fosters a greater sense of community and helps provide resources to those in need. We therefore hypothesize (H5) that voter turnout and volunteer time are positively related to HDI and National Happiness Index, and negatively related to income inequality.

## Methods

### Research Context: Global Development of Applied Community Studies Project

Our study is part of the ongoing, international collaborative GDACS project^1^, which uses the aggregated country-level survey, social and economic indicator data in the present study to predict and analyze the global growth of 12 applied community studies disciplines: community development, community psychology, community sociology, community social work, development anthropology, development economics, public health, urban/regional planning/geography, public administration/policy studies, popular/community education, liberation theology/faith-based community development studies, and interdisciplinary community research and action. The goal of the larger project is to assist countries in the development of their own indigenous applied community studies expertise and human resources.

### Selection of Countries

This study uses data from 105 states (103 nations and two territories) representing 95% of the world’s population. Countries were non-randomly selected based on population (all countries exceeding 10,000,000 population, excluding North Korea for lacking reliable data), with 20 smaller states (most 5–10 million population, but including the two territories: Palestine and Puerto Rico) added for purposes related to the GDACS Project (For more details on selection of countries in the GDACS database, see Hanitio and Perkins, 2017).

### Data and Measures

Data were gathered data from existing, publicly available databases. The predictor variables are all from 2009 to 2015 and the measures of national well-being are all from 2018 to 2020 to ensure that all predictors precede all well-being outcome measures in time and aid interpretation of results. Measures of well-being at the country level include the U.N. Human Development Index (HDI), income inequality (GINI), and National Happiness (U.N. Sustainable Development Solutions Network world survey of life satisfaction). The predictors of each measure of well-being include civil and political rights (Freedom House, 2015), a history of grassroots activism (Global Non-violent Action Database), citizen volunteering (Gallup Civic Engagement Index) and voter participation (Institute for Democracy and Electoral Assistance). Descriptive statistics (valid n, range, mean, and standard deviation] for each dependent and independent variable appear in Table 1 and a complete table of the values for each variable and all countries can be found in the Supplementary Appendix.

**TABLE 1:** Descriptive statistics: HDI, Net GINI, national happiness, grassroots activism, political decentralization, fiscal decentralization, freedom score, voter turnout, and volunteered time.

**Measure**	***n* ^a^**	**Range**	**Mean**	**SD**
HDI	102	0.394–0.957	0.72	0.16
Net Gini	78	0.249–0.577	0.38	0.07
National Happiness	97	2.567–7.809	5.49	1.17
Grassroots Activism	104	0.30–3.39	1.46	0.48
Political Decentralization	103	0–1	0.51	0.23
Fiscal Decentralization	103	0.06–1	0.38	0.23
Freedom Score	105	1–7	3.47	1.98
Voter Turnout	95	0.192–0.994	0.61	0.17
Volunteered Time	85	3–46	20.1	10.37

*^a^ Number of countries for which there were available data.*

### Dependent Variables: Measures of National Well-Being

To measure human development more holistically than the traditional focus on GDP in international development, we use the United Nations *Human Development Index*. HDI consists of per-capita Gross National Income (GNI; as a measure of economic well-being), average life expectancy (proxy for health well-being), and expected years of schooling (or educational attainment as a measure of capabilities well-being). HDI has been used extensively to study human development, is available for almost all countries, and again is a more comprehensive measure of well-being than those based solely on economic data. [Note: we also tested a fourth measure of well-being—Inequality-adjusted HDI (or IHDI)—which controls for the dispersion of each component of HDI, or the inequality of income, life expectancy, and educational attainment. Because IHDI correlated almost perfectly (*r* = 0.985) with HDI, we excluded those results from all tables but comment further on it in the Discussion].

To measure *income inequality*, which places even more emphasis on the distribution of economic benefits of a society, regardless of how relatively poor or wealthy that society is as a whole, we use *net Gini*, which is the mean Gini over 5 years (2014–2018). Gini coefficient is a measure of dispersion (variance) of values in the distribution of income in a population (or it can be calculated for wealth inequality). Gini coefficients theoretically range from 0 (perfect equality) to 1 (perfect inequality), but in practice, lower values are in the 20–40% range and higher values are in the 41–65% range. Gini is the most widely used measure of inequality, and net Gini provides stability to the measure by evening out year-to-year economic fluctuations.

To measure *happiness*, we use National Happiness Index, values for which are taken from the Statistical Appendix for Chapter 2 of the World Happiness Report (Helliwell et al., 2020). This measure is based on the U.N. Sustainable Development Solutions Network/Gallup World Poll telephone or face-to-face survey of life satisfaction data in which respondents rate their own subjective well-being. Nationally representative samples of generally 1,000–6,643 respondents per country (depending on population) respond to the Cantril life ladder: translated into all official languages of each country as needed, they are asked: “Please imagine a ladder, with steps numbered from 0 at the bottom to 10 at the top. The top of the ladder represents the best possible life for you and the bottom of the ladder represents the worst possible life for you. On which step of the ladder would you say you personally feel you stand at this time?” (Helliwell et al., 2020, Statistical Appendix p. 1). The NHI estimates national well-being of 153 countries comprising 99% of the world’s population, making it by far the largest and most reliable survey of national happiness.

### Social Justice Predictors of National Well-Being

#### Grassroots Activism

Number and success of past, non-violent grassroots action campaigns is a country-level measure we carefully coded using the Global Non-violent Action Database (Swarthmore College, 2015), the most comprehensive such database available, containing thousands of cases and summaries of historical grassroots social movement campaigns and mass actions across hundreds of countries. This database contains a Non-violent Action Product Score, which combines the number of cases in a country and weights them by their success rate. In this study, we use the base −10 log of this product score to adjust for positive skewness among the scores (namely the United States and other positive outliers; for more details, see Hanitio and Perkins, 2017).

#### Civil Liberties and Political Rights

For civil and political rights, we draw on Freedom House’s Civil Liberties (CL) and Political Rights (PR) indices from their annual Freedom in the World Report. This report represents a combination of surveys of residents and non-governmental organizations, review of news articles, and analysis by Freedom House staff. The indexes are based on “electoral process, political pluralism and participation, the functioning of the government, freedom of expression and of belief, associational and organizational rights, the rule of law, and personal autonomy and individual rights.” In this study, we average the 2015 CL and PR indices to create an overall “Freedom Score.” Freedom House’s survey of 210 countries and territories is the most widely used country-level measure of political rights and civil liberties (1–7 rating-scale). Due to the high correlation between these two variables (*r* = 0.98), this creates a single, simple measure that can be used to measure the presence of political rights and civil liberties. As PR and CL are coded at the source such that a lower score means more freedom, we reverse coded our Freedom Score so that higher values constitute more freedom.

#### Political Decentralization

It is one of several government decentralization indexes created by the World Bank (Ivanyna and Shah, 2012), which also includes fiscal (below), administrative, and other forms of decentralization in 182 countries. It is the only comprehensive set of national government decentralization measures. Political decentralization is measured based on three criteria: (1) election of legislative bodies and (2) executives in local governments by members of the community (rather than appointment by the central government) and (3) the extent to which local citizens are allowed to participate in decisions affecting their community.

#### Fiscal Decentralization

Similar to political decentralization, above, this is an index created by the World Bank of the level of public budgetary decision-making devolved from central to local discretionary authority (Ivanyna and Shah, 2012). Fiscal decentralization is calculated based on five variables: (1) degree of fiscal autonomy enabling local governments to engage in higher-level financing (e.g., selling bonds) to overcome fiscal gaps between expenditures and revenues; (2) ability of local governments to conduct their own taxation policies; (3) extent to which local governments can utilize unconditional or formula-based grants and transfers; (4) degree of autonomy of local governments in making spending decisions; (5) degree of autonomy in borrowing from external sources.

#### Citizen Volunteerism

Percent of citizens volunteering time to a service organization was taken from the Gallup Civic Engagement Index, which is based on Gallup’s regular international interview survey of more than 145,000 adults in 140 countries in 2009–2010. Respondents were asked whether they have done any of the following in the past month: donated money to a charity, volunteered time to an organization, or helped a stranger or someone they didn’t know who needed help. We used just the percent of the country’s sample who reported volunteer time, with higher scores indicating a higher level of civic engagement.

#### Voter Participation in Parliamentary Elections

To measure voter turnout, we use participation in parliamentary elections from 2009 to 2015 from the Institute for Democracy and Electoral Assistance global voter database. We considered averaging presidential and parliamentary turnout numbers in each country, but many countries have prime ministers elected by the parliament. As such, here we only use parliamentary voter participation data. One important caveat is that we use voter turnout as a percent of voting age population (VAP), not as a percent of registered voters. This is because ‘‘the roll is extremely difficult to keep up to date, and deaths or movements of electors from one district to another are not reflected in the roll, something which is a common problem facing electoral administrators around the world’’ (^2^, par. 5).

## Data Analyses

We begin by presenting descriptive statistics (mean, SD, and effective N) for each independent and dependent variable. We then examine Pearson correlations among each dependent (well-being) indicator, correlations among the various political, citizenship and social justice independent variables, and correlations between each independent and dependent variable. In addition, all correlations below 0.40 were scatterplotted to look for evidence of curvilinearity.

We conclude by testing a separate model for each dependent variable using hierarchical multiple linear regression (MLR) as the data structure is unnested and our sample size is too small for structural equation or other complex modeling. The MLR predicting each well-being dependent variable proceeds in three steps, starting with the most historical predictor grassroots activism, followed in step two by the three variables characteristic of a country’s political structure and so thought to be fairly stable (freedom score, political and fiscal decentralization), and in step three we add the individual behavioral (and so least stable) predictors volunteerism and voter participation. We will examine the degree to which each step as well as each independent variable predicts each measure of national well-being above and beyond the influence of the prior steps and other predictors in the model. We use listwise deletion of missing data in each MLR model.

## Results

### Correlations Among National Well-Being Indicators

Table 2 is a matrix of bivariate Pearson correlations among all independent and dependent variables. Beginning with just the indicators of national well-being, all three are significantly inter-related, confirming hypothesis H1. As expected, HDI (*r* = 0.79, *n* = 97, *p* < 0.001) is strongly positively related to national happiness. The bivariate results also confirm the expected negative correlations between Net GINI (with higher scores meaning more unequal distribution of income) and both HDI (*r* = −0.36, *n* = 78, *p*<0.001) and national happiness (*r* = −0.44, *n* = 78, *p* < 0.001) (see Table 2).

**TABLE 2:** Correlations among measures and predictors of national well-being.

	**1**	**2**	**3**	**4**	**5**	**6**	**7**	**8**	**9**
1. HDI	–	−0.36^***^	0.79^***^	0.64^***^	0.37^***^	0.21	0.36^***^	0.45^***^	0.65^***^
2. Income Inequality		–	−0.44^***^	−0.41^***^	0.02	−0.04	−0.07	−0.21	−0.26^*^
3. National Happiness Index			–	0.66^***^	0.36^***^	0.31^**^	0.32^**^	0.43^***^	0.60^***^
4. Freedom Score				–	0.14	0.24^*^	0.40^***^	0.50^***^	0.62^***^
5. Parl. Voter Turnout					–	0.01	−0.05	0.23^*^	0.15
6. Volunteered Time						–	0.20	0.20	0.16
7. Grassroots Activism							–	0.35^***^	0.53^***^
8. Political Decentralization								–	0.49^***^
9. Fiscal Decentralization									–

***p < 0.001, **p < 0.01, and *p < 0.05.

### Correlations Among Political, Citizenship and Social Justice Predictors

Most, but not all, hypothesized (H2) relationships among predictors depicted in Figure 1 were significant. A history of grassroots activism correlates positively with Freedom Score (*r* = 0.40, *n* = 103, *p* < 0.001), political decentralization (*r* = 0.35, *n* = 102, *p* < 0.001), and fiscal decentralization (*r* = 0.53, *n* = 102, *p* < 0.001), but only marginally with volunteered time (*r* = 0.20, *n* = 85, *p* = 0.063) and not at all with parliamentary voter turnout. Political decentralization correlates positively with Freedom Score (*r* = 0.50, *n* = 103, *p* < 0.001), fiscal decentralization (*r* = 0.49, *n* = 103, *p* < 0.001), and voter turnout (*r* = 0.23, *n* = 95, *p* < 0.05), but only marginally with volunteered time (*r* = 0.20, *n* = 85, *p* = 0.068). Fiscal decentralization correlates positively with Freedom Score (*r* = 0.62, *n* = 103, *p* < 0.001), but not significantly with voter turnout (*r* = 0.15, *ns*) or volunteered time (*r* = 0.16, *ns*). Contrary to expectations, Freedom Score correlated positively with volunteered time (*r* = 0.24, *n* = 85, *p* < 0.05) but not significantly with voter turnout. Volunteered time was unrelated to voter turnout, thus justifying our lack of hypothesis.

### Correlations of Political, Citizenship and Social Justice Predictors With National Well-Being

Examining the relationships between the predictors and each outcome variable, we found a significant correlation between freedom score and each measure of national well-being: HDI (*r* = 0.64, *n* = 102, *p* < 0.001), income equality (net GINI or *in*equality: *r* = −0.41, *n* = 78, *p* < 0.001), and happiness (*r* = 0.66, *n* = 97, *p* < 0.001). The other strong predictor was fiscal decentralization which correlated with HDI (*r* = 0.65, *n* = 101, *p* < 0.001), income inequality (*r* = −0.26, *n* = 78, *p* < 0.05), and national happiness (*r* = 0.60, *n* = 96, *p* < 0.001).

Political decentralization also correlated with HDI (*r* = 0.45, *n* = 102, *p* < 0.001) and national happiness (*r* = 0.44, *n* = 97, *p* < 0.001), but only marginally with income inequality (*r* = −0.21, *n* = 78, *p* = 0.064). There were also significant correlations between grassroots activism and both HDI (*r* = 0.36, *n* = 102, *p* < 0.001) and national happiness (*r* = 0.32, *n* = 97, *p* < 0.01). There was a positive correlation between voter turnout and HDI (*r* = 0.37, *n* = 95, *p* < 0.001) and national happiness (*r* = 0.36, *n* = 95, *p* < 0.001). Finally, we found a positive correlation between volunteered time and national happiness (*r* = 0.31, *n* = 85, *p* < 0.01).

We examined scatterplots of all correlations in Table 2 between −0.4 and +0.4 searching for any curvilinear relationships. No such relationships were found.

We also examined a predictor not depicted in Figure 1. Given the importance of political freedoms to national well-being, and the relationship of cultural “looseness” (tolerance for heterogeneous, or deviation from, values and norms of behavior) vs. “tightness” (CLT) to freedom, we wanted to consider the possible relationship of cultural looseness to well-being. We could only find CLT values (Uz, 2015) for 49 of our sampled countries and so did not include CLT in our model or multivariate analysis below. But we did find significant bivariate correlations for CLT with HDI (*r* = 0.629, *p* < 0.001), Net Gini (*r* = −0.325, *p* < 0.05), and NHI (*r* = 0.634, *p* < 0.001).

### Multiple Regression Prediction of National Well-Being Measures

We conducted three separate hierarchical multiple regressions to see how history of grassroots activism, political and fiscal decentralization, political rights and civil liberties, citizen volunteerism, and voter participation predict each form and measure of national well-being in a country. Each analysis included the same three steps. Using a chronological order in which more historical and stable variables entered the analysis earlier, we added grassroots activism in the first step, political and fiscal decentralization and freedom score in the second step, and parliamentary voter turnout and volunteered time in the final step.

The first analysis examining the influences of the six predictors on HDI revealed that grassroots activism alone significantly explained 15.5% of the variance in HDI in the first step (*p* < 0.001), whereas decentralization (mainly fiscal) and freedom score explained 43.2% additional variance after controlling for grassroots activism in the second step (*p* < 0.001). The final step showed that parliamentary voter turnout and volunteerism together explained 3.9% additional variance after controlling for other predictors (*p* < 0.05). The model as a whole explained about 60% of the total variance in a country’s HDI. Also, the standardized beta coefficients of fiscal decentralization, freedom score, and voter turnout were statistically significant, when all six predictors entered the analysis in the final step (see Table 3).

**TABLE 3:** Standardized beta coefficients and *R*^2^ increments^a^ in hierarchical multiple regressions predicting different measures of national well-being.

		**HDI**		**Income inequality**		**National happiness**	
**Step**	**Independent variables:**	**Final beta**	***R*^2^ Δ**	**Final beta**	***R*^2^ Δ**	**Final beta**	***R*^2^ Δ**
1	Grassroots Activism	0.025	0.155^***^	0.136	0.011	0.061	0.148^***^
2	Political Decentral.	0.063	0.432^***^	−0.081	0.154^*^	−0.022	0.354^***^
	Fiscal Decentral.	0.403^***^		−0.076		0.315^**^	
	Freedom Score	0.326^**^		−0.403^*^		0.360^**^	
3	Voter Turnout	0.208^**^	0.039^*^	0.126	0.019	0.203^*^	0.062^*^
	Volunteered Time	0.034		0.071		0.175^*^	
		*R* ^2^	Adjusted *R*^2^	*R* ^2^	Adjusted *R*^2^	*R* ^2^	Adjusted *R*^2^
	Full Model	0.626	0.594	0.185	0.104	0.564	0.525

*^a^We added grassroots activism in the first step, political decentralization, fiscal decentralization, and freedom score in the second step, and parliamentary voter turnout and volunteered time in the final step.*

***p < 0.001, **p < 0.01, and *p < 0.05.

The second analysis found that grassroots activism explained barely over 1% (ns) of the variance in Net GINI in the first step. In the second step, political and fiscal decentralization and freedom score explained 15.4% additional variance after controlling for grassroots activism, which was statistically significant (*p* < 0.01). Finally, parliamentary voter turnout and volunteerism explained 1.9% (ns) additional variance after controlling for other predictors. The model as a whole explained less than 19% of the total variance in a country’s income inequality. Only the standardized beta coefficient of freedom score (*b* = −0.403, *p* < 0.001) was statistically significant, when all predictors entered the analysis in the final step (Table 3).

The final analysis that examined the influences of the six predictors on national happiness showed that grassroots activism significantly explained 14.8% of the variance in national happiness in the first step (*p* < 0.001). In step two, freedom score and fiscal decentralization (with political decentralization negligibly contributing) explained 35.4% additional variance after controlling for grassroots activism (*p* < 0.001). Lastly, parliamentary voter turnout and volunteerism explained 6.2% additional variance after controlling for other predictors (*p* < 0.05). The model as a whole explained over 50% of the total variance in the happiness or life satisfaction of a country. The standardized beta coefficients of freedom score, fiscal decentralization, voter turnout, and volunteer time were all statistically significant in the full final model (Table 3).

**FIGURE 2:**
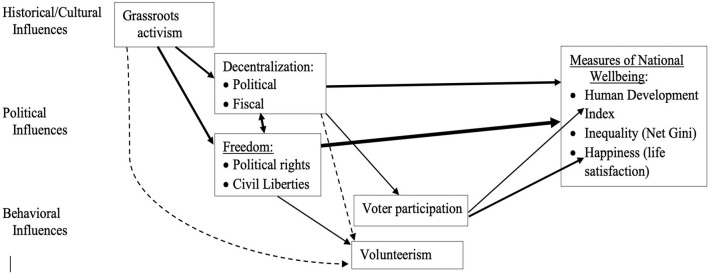
Confirmed Political, Social Justice, and Citizenship Predictors and Measures of National Well-being.

Taken together, Tables 2, 3 confirm many, but not all, of the political, social justice, and citizenship predictors of each other and of our three measures of national well-being (see Figure 2).

## Discussion

This study had two main goals. The first was to simultaneously examine a more comprehensive range of international indicators of national well-being beyond just economic wealth or income, including HDI (with its focus on life expectancy and years of schooling in addition to per capita income), income equality, and Happiness Index or life satisfaction. Those three indicators provide a range of widely accessible measures of different kinds of human development or well-being. Their significant intercorrelation provides cross-validation, but income inequality was less closely aligned with the other two measures of well-being, as reflected for example by the United States having among the highest development and happiness scores but also a moderately high Net Gini, with as much, or even more, income inequality as Mali and many other very low-HDI and less happy countries have (see Supplementary Appendix).

The second goal was to propose and test a new model of the political, social justice, and citizenship factors that predict well-being. Support for the proposed model was mixed. By far the strongest predictors of HDI and happiness were Freedom Score and fiscal decentralization. Countries with more and better protected political rights and civil liberties and with more local control of the public purse are more developed in terms of life-expectancy, education, and income, and also happier or more satisfied with life. Freedom is also the primary predictor of income equality in our model. Those results clearly support the Diener et al.’s (1995) finding that subjective well-being was most consistently related to a country’s culture of liberal individualism (also consistent with our own correlational results for cultural “looseness”), Sen’s (1999) theory of “development as freedom,” and partly confirms Frey and Stutzer’s (2000) findings that “happiness prospers in democracy.” Our data suggest the connection is more about political and civil rights, local budgetary control and, to a lesser extent, voter participation than it is about political decentralization, activism, or volunteerism. It may also be the case that the causal relationship is bidirectional—although our predictors were generally measured about 5 years before the well-being measures, both tend to be fairly stable, so it is possible that over the long term, greater economic and social development, equality, and life satisfaction enables political leaders to grant more freedoms to citizens.

Although less predictive than political and civil rights and fiscal decentralization, grassroots activism, political decentralization, and voter turnout in parliamentary/congressional elections also had significant positive correlations with HDI and Happiness, but not with income inequality. However, of those three, only voter turnout remained modestly significant in the multiple regression analyses. We acknowledge that the causal path from more education, lifespan and income to higher voter participation may be at least as likely as higher congressional/parliamentary election turnout providing better law-makers and policies that lead to those developmental benefits. However, we think the link between 2009 and 2015 turnout and life satisfaction surveyed in 2019 supports prior evidence that greater democratic participation leads to more well-being (Frey and Stutzer, 2000, 2010; Inglehart and Klingemann, 2000), but may also partly reflect voters reporting higher life satisfaction in order to justify their vote or avoid the cognitive dissonance (Festinger, 1957) of having chosen ineffective leaders.

Significant suppression effects were observed due to Freedom Score’s substantial correlation with activism and political decentralization, suggesting any effect activism and political decentralization have on well-being is mainly through their influence on increasing political and civil rights (Sen, 1999; Inglehart and Klingemann, 2000). This makes it all the more noteworthy that fiscal decentralization remained such a strong predictor of HDI and happiness even after controlling for the influence of freedom score, which is fiscal decentralization’s strongest correlate among predictors.

One area requiring much more measurement and analytical work is the role of inequality in development and national well-being. We say that not only because inequality is such a drag in so many ways on human development and wellness (Wilkinson and Pickett, 2009) and not only because our model was most limited in predicting income inequality. We conclude that because we also analyzed Inequality-adjusted HDI as a separate measure of national well-being. It is an indicator formulated to account for the effects of inequality on development or, more precisely, for the uneven distributional inequalities of income, education, and longevity in a population (Hicks, 1997). We did not report the results here, however, because IHDI correlated so highly with HDI, the prediction models were nearly the same. This suggests that reformulating IHDI might make it a more independent and useful measure of human development in the context of health, education, and income inequality. There are also more recent alternative measures: for example, Bilbao-Ubillos (2013) added indicators for gender equality, income inequality, and personal safety to create the Composite Dynamic Human Development Index.

### Limitations and Future Research

We expected that countries with more volunteerism would be more developed, equal, and happy. Those are common goals of volunteering, but we also understood that development problems, inequality, and life dissatisfaction create the need for more voluntary service activity. However, volunteerism was not significantly related to the development or inequality measures of national well-being. It did correlate with life satisfaction or happiness, but even that became a non-significant trend in the multiple regression. The lack of significant unique well-being effects of volunteerism at the national level may be due to the simple measure of asking survey respondents whether or not in the past month they volunteered time to an organization or it may be due to the considerable international skew in organized volunteerism: it is common in some countries, including the United States, but in many countries mutual assistance or “neighboring” occurs informally and organized volunteering is uncommon. Both more research and better measurement of different forms of mutual aid activity and its effects on well-being—not just of providers and recipients, but of communities and countries as a whole—are needed.

The use of national indicator data inevitably raises validity questions. Some we chose, such as Freedom Score and Fiscal and Political Decentralization, are based on rigorous and complex, multi-dimensional assessment and multiple sources. Besides volunteerism, however, others were represented by the most relevant and universal indicators we could find, but are based on relatively crude proxy measures. For example, HDI is the mostly widely used measure of international social and economic development, but each of its three components has limits based on the necessary reliance on reliable data collected by virtually every country. That is of particular concern regarding its inclusion of life expectancy, which is only a rough proxy for population health and quality healthcare access. National Happiness is also based on a single representative survey question about overall life satisfaction. Grassroots Activism was less predictive of well-being than we expected based on prior research using the same measure significantly and robustly predicting countries’ strength of community development and related professional resources (Hanitio and Perkins, 2017; Lyew et al., 2021). The limits of the non-violent activism measure have mainly to do with its skew favoring United States cases, which is why we statistically adjusted it to reduce the effect of outliers. Despite this limitation, it is by far the most comprehensive global database of social movement activism we have found and contains thousands of cases and summaries of historical grassroots campaigns across hundreds of countries, collected and coded by hundreds of trained volunteers over several years.

Besides measurement of some of our variables, another limitation of this study is the simplicity of our correlation and regression analyses. Although obtaining adequate nested international data would be a challenge, a much stronger approach would be to use multilevel analyses of individual variation within local communities and how those vary within countries and then to partition variance at those levels in comparing country-level variation. Use of qualitative or mixed methods would also greatly strengthen our analysis and conclusions.

Although we purposely sequenced the timing of predictor variables measurement to precede collection of well-being outcome measures, there is still a chance that global historical events, such as the economic crisis that preceded the (2009–2015) predictors or the COVID-19 pandemic at the end of the (2016–2020) outcome measurement period, may have influenced either of those periods and thus be a threat to validity.

Our main findings are that a history and culture of non-violent grassroots activism predict political freedoms and government decentralization, and that freedom and fiscal decentralization, along with voter participation, in turn significantly predict later national well-being. Each of those relationships warrant further comparative psychological as well as political, sociological, anthropological, and economic research. For example, we were unable to find valid and reliable data on corruption in 80% of our sampled countries, but future research should aim to analyze the influence of corruption (in the context of other predictors) on HDI, income inequality, and happiness. Also, precisely how and why do critical hopefulness (Christens et al., 2013), voting (Avery, 2015), and more collective activist behaviors (Hanitio and Perkins, 2017) vary among countries globally? How do people think about their freedoms and how those relate cognitively and behaviorally to their well-being (Kosher and Ben-Arieh, 2017)? And why do the “objective” human development indicators relate to both greater equality and a subjective sense of wellbeing and life satisfaction more closely in some countries than others?

The importance of cultural looseness-tightness (CLT) also deserves more attention (Uz, 2015). In bivariate analyses, cultural looseness—or tolerance for deviations from value and behavioral norms—was nearly as predictive of all four measures of well-being as was Freedom Score. That makes sense as the absence of strict norms (and the pressure to adhere to them) allows people to live more fulfilling lives. Unfortunately, CLT scores have not been calculated for enough countries to include in the multivariate analysis. Yet its strong positive correlation with multiple well-being variables warrants further research into this indicator as well as other cultural variables and their effects on well-being.

## Conclusion

Our findings extend Van (2017) and other studies by adding independent variables more focused on political freedoms and social justice and citizenship behaviors and diversifying the dependent variables used to create a more complete picture of national well-being and what leads to it. We hope the present findings and proposed further research may aid the creation of effective policies to increase both objective and subjective well-being around the globe. If political rights and civil liberties are the dominant predictors of national well-being, it is vital that those freedoms be established where they currently are not and protected everywhere. Based on our prior analyses in the GDACS project, a significant and surprisingly under-researched challenge is the fact that, globally, applied psychological and other research and professional human resources are weakest precisely where they are most needed (Hanitio and Perkins, 2017; Lyew et al., 2021; Ozgurer and Perkins, 2021). Programs and policies to effectively address social justice, citizenship, well-being, inequality, human rights, and other development challenges must be based on locally sourced, valid and reliable information and applications. Thus, indigenous undergraduate and graduate programs, research and dissemination, and professional associations and other resources must be developed to work closely with and support local community health, education, and political and human rights organizations in countries where they are needed. As Lyew et al. (2021) found, any assistance wealthy countries or individuals provide must not be based on counterproductive foreign aid models or dictates.

## Data Availability Statement

The data presented in the study are included in the article/Supplementary Material. Further inquiries can be directed to the corresponding author/s.

## Author Contributions

DP supervised all the research, lead conceptualizer, interpretation of results, edited all the sections of the manuscript, and created figures. MO helped to identify and gather the data, conducted the final analyses, and drafted most of the Results and Tables. AL helped to identify and gather the data, conducted the preliminary analysis, and drafted portions of the Literature Review, Methods, and Supplementary Appendix. SO-T drafted portion of the Literature Review. All authors contributed to the article and approved the submitted version.

## Conflict of Interest

The authors declare that the research was conducted in the absence of any commercial or financial relationships that could be construed as a potential conflict of interest.

## Publisher’s Note

All claims expressed in this article are solely those of the authors and do not necessarily represent those of their affiliated organizations, or those of the publisher, the editors and the reviewers. Any product that may be evaluated in this article, or claim that may be made by its manufacturer, is not guaranteed or endorsed by the publisher.

## Footnotes

1
https://www.researchgate.net/project/Global-Development-of-Applied-Community-Studies

2
https://www.idea.int/data-tools/data/voter-turnout
