# Supplementary material for: Well-Being as Human Development, Equality, Happiness and the Role of Freedom, Activism, Decentralization, Volunteerism and Voter Participation: A Global Country-Level Study
Source: Front Psychol. 2021 Sep 17;12:745818. doi: 10.3389/fpsyg.2021.745818 (PMC8484634; doi:10.3389/fpsyg.2021.745818)
Supplement: Supplementary file 1 [file Data_Sheet_1.pdf]

Appendix: Countries and Country-level Values for Each Variable

Country	2019 HDI	2018 Net Gini	2020 NHI	2015 Freedom Score	2011-2015 Parl. Voter Turnout (%VAP)	2009-2010 % Volunteers	2011 Base-10 log NVA prod. score	2012 Pol. Decentralization	2012 Fiscal Decentralization
Afghanistan	0.511		2.567	6	29.71%		0.954	0	0.18
Algeria	0.748	0.322	5.005	5.5	38.70%	5	1.362	0.28	0.24
Angola	0.581			5.5	71.59%		1.000	0	0.14
Argentina	0.845	0.386	5.975	2	78.95%	16	1.699	0.75	0.53
Australia	0.944	0.332	7.223	1	78.86%	36	2.070	0.67	0.78
Barbados	0.814			1	69.78%		0.699	0	0.06
Bangladesh	0.632	0.395	4.833	4	48.54%	14	1.580	0.25	0.45
Bolivia	0.718	0.435	5.747	3	86.19%	20	2.033	0.71	0.4
Brazil	0.765	0.449	6.376	2	75.09%	14	2.049	0.83	0.78
Bulgaria	0.816	0.339	5.102	2	60.88%	5	1.732	0.75	0.32
Burkina Faso	0.452		4.769	4.5	36.45%	10	1.130	0.50	0.08
Burma/Myanmar	0.583		4.308	6	61.87%		1.380	0	0.09
Cambodia	0.594	0.366	4.848	5.5	69.76%	10	1.477	0.33	0.2
Cameroon	0.563	0.398	5.085	6	37.55%	14	1.431	0.42	0.12
Canada	0.929	0.312	7.232	1	58.14%	34	2.594	0.83	0.9
Chad	0.398	0.383	4.423	6.5	57.27%	21	0.398	0.39	0.1
Chile	0.851	0.459	6.228	1	53.66%	16	1.881	0.50	0.57
China	0.761	0.51	5.124	6.5		4	2.208	0.25	0.57
Colombia	0.767	0.489	6.163	3.5	45.88%	24	1.806	0.67	0.55
Costa Rica	0.81	0.457	7.121	1	64.35%	24	1.653	0.31	0.49
Cuba	0.783			6.5	88.75%		1.519	0.67	0.28
Czech Republic	0.9	0.256	6.911	1	60.03%	18	1.230	0.58	0.73
Denmark	0.94	0.253	7.646	1	81.09%	24	1.447	0.58	0.9
Dem.Rep.Congo	0.48		5.194	6	55.56%	14	1.146	0.17	0.12
Dominican Rep.	0.756	0.446	5.689	2.5	58.57%	33	1.130	0.58	0.14
Ecuador	0.759		5.925	3	94.29%	15	1.884	0.67	0.44
Egypt	0.707	0.47	4.151	5.5	41.23%	8	1.816	0.33	0.1
El Salvador	0.673	0.389		2.5	61.23%	15	1.818	0.42	0.39
Ethiopia	0.485		4.186	6	71.17%		1.114	0.50	0.36
Finland	0.938	0.256	7.809	1	72.96%	30	1.491	0.67	0.76
France	0.901	0.299	6.664	1	50.73%	27	2.088	0.58	0.75
Germany	0.947	0.29	7.076	1	66.07%	26	2.124	0.75	0.67
Ghana	0.611	0.383	5.148	1.5	82.05%	30	1.538	0.33	0.41
Greece	0.888	0.332	5.515	2	70.01%	3	1.371	0.83	0.34
Guatemala	0.663	0.445	6.399	3.5	66.30%	33	1.736	0.33	0.18
Guinea	0.477		4.949	5	62.99%		1.301	0.21	0.22

Country	2019 HDI	2018 Net Gini	2020 NHI	2015 Freedom Score	2011-2015 Parl. Voter Turnout (%VAP)	2009-2010 % Volunteers	2011 Base-10 log NVA prod. score	2012 Political Decentralization	2012 Fiscal Decentralization
Haiti	0.51		3.721	5	19.28%	31	1.519	0	0.06
Hong Kong	0.949	0.54	5.510	3.5	53.05%	16	0.602	0.67	1
Hungary	0.854	0.287	6.000	2	63.36%	8	1.447	0.75	0.62
India	0.645	0.479	3.573	2.5	70.29%	18	2.326	0.67	0.48
Indonesia	0.718	0.457	5.286	3	82.93%	13	1.505	0.53	0.5
Iran	0.783	0.388	4.672	6	61.81%		1.906	0.21	0.6
Iraq	0.674		4.785	6	76.81%	7	1.342	0.50	0.06
Ireland	0.955	0.303	7.094	1	63.78%	38	1.204	0.58	0.27
Israel	0.919	0.369	7.129	1.5	74.65%	27	1.892	0.67	0.2
Italy	0.892	0.333	6.387	1	68.33%	14	1.826	0.83	0.49
Jamaica	0.734		5.890	2.5	40.98%		0.301	0.50	0.22
Japan	0.919	0.299	5.871	1	59.67%	28	1.699	1	0.68
Kazakhstan	0.825	0.288	6.058	5.5	56.88%	25	1.000	0.33	0.37
Kenya	0.601	0.416	4.583	4	54.99%		1.740	0.33	0.36
Lebanon	0.744		4.772	4.5	66.34%		1.000	0.50	0.21
Madagascar	0.528	0.372	4.166	4	35.29%		1.279	0.75	0.13
Malawi	0.483	0.373	3.538	3.5	50.90%	35	1.000	0.67	0.13
Malaysia	0.81	0.428	5.384	4	62.95%	20	1.204	0.08	0.41
Mali	0.434	0.323	4.729	4.5	35.12%	14	1.279	0.44	0.11
Mexico	0.779	0.459	6.465	3	56.32%	25	1.708	0.83	0.42
Morocco	0.686	0.357	5.095	4.5	28.65%		1.550	0.50	0.26
Mozambique	0.456	0.399	4.624	3.5	44.87%		1.301	0.17	0.41
Nepal	0.602	0.395	5.137	3.5	57.36%	22	1.380	0.75	0.22
Netherlands	0.944	0.266	7.449	1	71.02%	37	1.568	0.50	0.36
New Zealand	0.931	0.325	7.300	1	71.09%	39	1.869	0.67	0.79
Nicaragua	0.66	0.432	6.137	3.5	71.84%	17	1.079	0.75	0.16
Niger	0.394		4.910	3.5	45.64%	11	0.954	0.39	0.44
Nigeria	0.539	0.39	4.724	4.5	25.80%	41	1.740	0.67	0.24
Norway	0.957	0.249	7.488	1	77.93%		1.695	0.58	0.74
Pakistan	0.557	0.362	5.693	4.5	40.43%	27	1.732	0.56	0.5
Palestine	0.708		4.553	5.5		9	1.740	0.25	0.34
Panama	0.815	0.461	6.305	2	79.03%	24	1.021	0.33	0.2
Paraguay	0.728	0.449	5.692	3	58.15%	35	0.954	0.67	0.39
Peru	0.777	0.454	5.797	2.5	87.41%	19	1.806	0.75	0.37
Philippines	0.718	0.479	6.006	3	69.45%	41	1.740	0.75	0.5
Poland	0.88	0.321	6.186	1	48.97%	13	1.775	0.58	0.62
Portugal	0.864	0.348	5.911	1	63.12%	8	0.699	0.75	0.56

Country	2019 HDI	2018 Net Gini	2020 NHI	2015 Freedom Score	2011-2015 Parl. Voter Turnout (%VAP)	2009-2010 % Volunteers	2011 Base-10 log NVA prod. score	2012 Political Decentralization	2012 Fiscal Decentralization
Romania	0.828	0.33	6.124	2	42.81%	5	1.204	0.58	0.43
Russia	0.824	0.439	5.546	6	57.96%	26	1.681	0.71	0.34
Rwanda	0.543	0.443	3.312	6	99.38%	11	0.602	0.53	0.08
S. Africa	0.709	0.577	4.814	2	53.77%	14	2.061	0.42	0.58
S. Korea	0.916	0.307	5.872	2	42.47%	27	2.188	0.75	0.70
S. Sudan	0.433		2.817	6.5			1.255		
Saudi Arabia	0.854		6.406	7		26	0.602	0.08	0.06
Senegal	0.512	0.348	4.981	2	30.27%	10	1.740	0.42	0.24
Spain	0.904	0.343	6.401	1	63.83%	18	1.820	0.50	0.74
Sri Lanka	0.782	0.514	4.327	5	76.55%	46	0.903	0.47	0.22
Sudan	0.51			7	64.11%	23	1.255	0.39	0.22
Sweden	0.945	0.257	7.353	1	82.61%	14	1.659	0.54	0.77
Syria	0.567			7	20.28%	10	1.312	0.25	0.16
Taiwan			6.455	1.5	74.22%	19	0.845	0.67	0.56
Tanzania	0.529	0.422	3.476	3	0.3753	18	1.176	0.5	0.21
Thailand	0.777	0.437	5.999	5.5	54.32%	16	1.740	0.58	0.46
Trinidad/Tobago	0.796		6.192	2	78.10%		1.146	0.50	0.11
Tunisia	0.74	0.333	4.392	2	49.65%	6	1.000	0.50	0.34
Turkey	0.82	0.398	5.132	3.5	0.8184	7	1.760	0.58	0.46
Uganda	0.544	0.376	4.432	5.5	55.32%	23	1.342	0.75	0.2
UK	0.932	0.328	7.165	1	60.45%	28	2.367	0.67	0.52
Ukraine	0.779	0.263	4.561	3		30	1.531	0.64	0.35
Uruguay	0.817	0.37	6.440	1		12	1.342	0.83	0.55
USA	0.926	0.378	6.940	1	42.39%	43	3.395	1	0.9
Uzbekistan	0.72		6.258	7	96.93%		0.477	0.53	0.18
Venezuela	0.711		5.053	5	66.60%	11	1.398	0.67	0.48
Vietnam	0.704	0.422	5.353	6	98.97%	12	0.845	0.58	0.16
Yemen	0.47		3.527	6		5	1.146	0.17	0.08
Zambia	0.584	0.495	3.759	3.5	42.25%	24	1.255	0.25	0.31
Zimbabwe	0.571	0.398	3.229	5.5		21	1.230	0.42	0.17
